# Open vs. robot-assisted laparoscopic ureteral reimplantation in a contemporary pediatric cohort: a retrospective single-institution analysis

**DOI:** 10.1007/s11701-026-03169-z

**Published:** 2026-02-05

**Authors:** Suhaib Abdulfattah, Nicole J. Kye, Sanjay Aiyar, Emily Ai, Marina Quairoli, Meghan F. Davis, Karl Godlewski, Katherine Fischer, Christopher J. Long, Dana A. Weiss, Aseem R. Shukla, Arun K. Srinivasan, Sameer Mittal

**Affiliations:** 1 Division of Urology, Children’s Hospital of Philadelphia, Philadelphia, PA USA; 2 Division of Urology, Perelman Center for Advanced Care, Hospital of the University of Pennsylvania, Philadelphia, PA USA; 3 The Hub for Clinical Collaboration, Children’s Hospital of Philadelphia, 9th Floor 3500 Civic Center Blvd, Philadelphia, PA 19104 USA

**Keywords:** Vesicoureteral reflux, Ureteral reimplantation, Ralur, Open ureteral reimplant

## Abstract

Ureteral reimplantation (UR) in children is traditionally performed to correct vesicoureteral reflux (VUR). The traditional open approach can be performed using various techniques. More recently, the robotic approach has gained popularity but with concerns regarding cost, complications, and overall efficacy. This study aims to understand clinical outcomes in patients undergoing UR via both approaches. We hypothesize robot-assisted ureteral reimplantation (RALUR) has comparable outcomes to open ureteral reimplant (OUR) in a contemporary cohort. An IRB approved registry was queried for all patients with primary VUR who underwent UR procedures between 2015 and 2022. Demographics, pre/postoperative history, and operative details were retrospectively aggregated. There were 185 patients who met inclusion criteria: 44 OUR and 141 RALUR. Both cohorts had a similar rate of reported preoperative febrile UTI (91% OUR vs. 97% RALUR, p = 0.22). OUR cohort were younger, with a median age of 26.74 months versus 66.16 months in the RALUR group (p < 0.001) and had higher grades of VUR (p < 0.001). In terms of 30-day complications: Clavien-Dindo grade 3b events were reported in 2 RALUR patients, both requiring stent placement due to obstruction. During a median follow up of 21 months in the OUR and 16 months in the RALUR group (p = 0.68), both cohorts had similar rates of febrile UTI following surgery, with 7 (16%) patients in the OUR compared to 29 (21%) in the RALUR group (p = 0.66). Three patients had persistent VUR on postoperative VCUG that was managed with subureteral injection. In this single-institution cohort of contemporary patients undergoing UR, the RALUR approach had similar efficacy, complications and post-operative febrile UTI rate compared to the traditional open approach.

## Introduction

Vesicoureteral reflux (VUR) is a prevalent etiology in pediatric urology, with treatment options ranging from conservative management with continuous antibiotic prophylaxis (CAP) to surgical intervention, specifically ureteral reimplantation (UR) [1]. The open ureteral reimplantation (OUR) has traditionally been considered the gold standard approach, reporting success rates nearing 98% [2]. Newer approaches including robot-assisted ureteral reimplantation (RALUR) have emerged [3, 4], aiming to reduce morbidity while preserving surgical success. Surgical success can be evaluated radiographically through resolution of reflux on voiding cystourethrogram (VCUG) and/or clinically by the absence of postoperative febrile urinary tract infections (UTIs) [5, 6].

RALUR gained prominence by offering promising outcomes, including a short learning curve, improved ergonomics, 3D visualization, reduced opioid consumption, and shorter hospital stays compared to laparoscopic ureteral reimplantation (LUR) and OUR [7–9]. Previous comparative studies have been reported comparing the different surgical modalities for treating VUR [10, 11]. However, the debate over the gold standard approach for VUR management persists.

Our study aims to fill in the gaps by providing a multi-surgeon, single institution experience comparing OUR and RALUR. Specifically, we focus on relevant endpoints, including febrile UTI (f-UTI) following UR, complication rates, and need for a surgical reintervention. We hypothesize that RALUR demonstrates comparable outcomes to OUR.

## Methods

An IRB approved single institutional registry was utilized to retrospectively identify children who underwent a ureteral reimplantation (UR) procedure between January 2015 and December 2022. Inclusion criteria included children diagnosed with primary VUR. Children with underlying abnormalities, such as duplex collecting systems, neurogenic bladder, ureterocele, posterior urethral valve, ectopic ureter, bladder diverticulum, exstrophy-epispadias complex and primary obstructive megaureter (POM) were excluded. Children treated with subureteral injection prior to UR were also excluded.

The cohort was then stratified into 2 groups, OUR and RALUR, depending on the approach during surgical management. Both OUR and RALUR were performed throughout the study period, with both approaches offered concurrently at our institution. The surgical approach was determined based on surgeon preference and shared decision-making. Our RALUR technique aligns with previously published methods [12]. All robot-assisted procedures were done using the Da Vinci Surgical System (Intuitive Surgical, Inc., Sunnyvale, CA). OUR was performed using standard techniques at the discretion of the surgeon, including Cohen Cross-Trigonal, Glenn-Anderson, Politano-Leadbetter, and Lich-Gregoir approaches. Postoperatively, patients in both cohorts typically had an indwelling Foley catheter placed intraoperatively which was removed on postoperative day 1 followed by a trial of void prior to discharge, unless clinically indicated otherwise.

Baseline demographics and preoperative details including history of UTI, antenatal hydronephrosis, preoperative hydronephrosis, VUR severity based on the International Reflux Study in Children [13], constipation, and voiding dysfunction. History of UTI included both f-UTI and breakthrough UTI, f-UTI while on CAP. Voiding dysfunction was only assessed in toilet-trained patients based on parental observations of symptoms such as urinary frequency, urgency, and/or bladder-holding maneuvers. Intraoperative details included operative time (skin-to-skin), postoperative narcotic use, and length of hospital stay. Postoperative details encompassed 30-day complications, identified and graded based on the Clavien-Dindo classification [14], as well as postoperative febrile UTI, and reintervention procedures. Postoperative febrile UTI was defined as the presence of fever > 100.4 °F, associated with lower urinary tract symptoms (LUTS) or flank pain, pyuria on urinalysis, and a positive urine culture (≥ 100,000 colony-forming units [CFU] of a single uropathogenic organism). Urine specimens were obtained via clean catch, catheterization, or bag specimen. Postoperative VCUG was not routinely performed in all patients. Instead, it was offered based on clinical indications, like postoperative febrile UTIs. This institutional practice reflects a shift towards minimizing invasive imaging and radiation exposure in children, with a preference of clinical follow-up in the absence of symptoms.

Categorical variables were reported as frequency (percentage) while continuous variables were reported by the median and interquartile range. Statistical comparisons between the OUR and RALUR groups were conducted using Fisher’s exact test for categorical variables and the Wilcoxon rank-sum test for continuous variables. A multivariable logistic regression model was performed to evaluate the association between surgical approach and postoperative febrile UTI, while adjusting for age at surgery and VUR grade (classified as non-dilating [VUR grades I-II] and dilating [VUR grades III-V]). P values were two sided and a p value < 0.05 was considered statistically significant. All analysis was done using the statistical software Stata BE 18.0 (**Stata** Corp, College Station, TX, USA).

## Results

Our study included 185 patients—44 (24%) underwent OUR and 141 (76%) underwent RALUR. Females comprised 162 (88%) and males 23 (12%) of the cohort. Antenatal hydronephrosis was significantly more reported in the OUR cohort compared to RALUR (41.9% vs. 14.5%, *p* < 0.001).

Preoperatively, febrile UTI was present in 40 (91%) patients in the OUR group compared to 136 (97%) patients in the RALUR group (*p* = 0.22). Constipation was reported in 13 (29.5%) patients in the OUR group versus 69 (48.9%) in the RALUR group (*p* = 0.03), and voiding dysfunction in 6 (13.6%) versus 34 (24.3%), respectively (*p* = 0.15). The OUR cohort had a higher proportion of severe reflux (Grades IV-V) on voiding cystourethrogram (VCUG) compared to RALUR (73% vs. 40%, *p* < 0.001). The OUR group had a lower median age at the time of surgery compared to RALUR (2.23 years [IQR 1.42, 3.98] vs. 5.51 years [IQR 3.03, 8.99], *p* < 0.001) (Table 1).

**Table 1:** Baseline demographics and preoperative details comparing open ureteral reimplant (OUR) versus robot-assisted ureteral reimplant (RALUR)

	OUR	RALUR	
*N* = 185	44 (24%)	141 (76%)	
**Gender, n (%)** Male Female	8 (18.2) 36 (81.8)	15 (10.6) 126 (89.4)	0.20
Age (years)	2.23 (1.43, 3.98)	5.51 (3.03, 8.99)	< 0.001*
Antenatal hydronephrosis, n (%)	18 (41.9)	20 (14.5)	< 0.001*
Hydronephrosis on RBUS, n (%)	20 (45.5)	42 (29.8)	0.07
**SFU grade, n (%)** I II III IV	1 (2.2) 5 (11.4) 4 (9.2) 10 (22.7)	5 (3.5) 17 (12.1) 11 (7.8) 9 (6.4)	0.16
**VUR grade, n (%)** I II III IV V	0 (0) 1 (2) 11 (25) 17 (39) 15 (34)	2 (1) 23 (16) 61 (43) 40 (29) 16 (11)	< 0.001*
History of breakthrough UTI, n (%)	27 (61)	67 (49)	0.17
Febrile UTI, n (%)	40 (91)	136 (97)	0.22
DMSA scan, n (%)	20 (45.5)	77 (54.6)	0.30
Renal scar, n (%)	13 (29.5)	62 (43.9)	0.22
Constipation, n (%)	13 (29.5)	69 (48.9)	0.03
Voiding dysfunction^T^, n (%)	6 (13.6)	34 (24.3)	0.15

*p < 0.05

^T^ Voiding dysfunction was reported only in toilet trained children at the time of surgery. This entailed symptoms of urinary frequency, urgency, and/or bladder holding habits as reported by the parents and/or patient

SFU = society of fetal urology; VUR = vesicoureteral reflux

Estimates were reported as median (interquartile 1, interquartile 3) or frequency (percentage)

Patients with a low reflux grade (VUR Grade I-II, *n* = 26) underwent UR at a median age of 8.66 years (IQR 5.61, 12.05) compared to patients with higher-grade reflux (VUR grades III-V), who underwent surgery at a median of 3.7 years (IQR 2.13, 7.05; *p* < 0.001).

Among patients undergoing OUR (*n* = 44), the following techniques were used: Cohen Cross-Trigonal (50%), Lich-Gregoir (25%), Glenn-Anderson (23%), and Politano-Leadbetter (2%).

Perioperatively, 30 (68.2%) patients in the OUR and 81 (57%) in the RALUR group underwent bilateral procedures (*p* = 0.43). The OUR group had longer median operative times (222 min [IQR 186–251] vs. 193 min [IQR 169–229], *p* = 0.01) and hospital stay (2 days [IQR 1–3] vs. 1 day [IQR 1–2], *p* < 0.001). No significant difference was noted in terms of postoperative narcotic use represented by IV morphine equivalence—median of 0.01 mg/kg/day (IQR 0-0.13) in the OUR group vs. 0.01 mg/kg/day (IQR 0-0.05) in the RALUR group (*p* = 0.15).

Postoperatively, there was no significant difference in the 30-day complication rate between the groups (18% OUR vs. 10% RALUR, *p* = 0.18). Two patients who underwent bilateral UR, 1 OUR and 1 RALUR, experienced postoperative urinary retention that was managed by catheterization—a foley catheter was kept for 10–14 days after which they were able to void spontaneously. Clavien-Dindo grade 3b complications were reported only in 2 (1.3%) patients in the RALUR group, both requiring stent placement due to obstruction. One of these patients subsequently underwent an additional ipsilateral dismembered ureteral reimplantation due to unresolved obstruction (Table 2).

**Table 2:** Intraoperative, postoperative, and long-term outcomes comparing open ureteral reimplant (OUR) versus robot-assisted ureteral reimplant (RALUR)

	OUR	RALUR	
*N* = 185	44 (24%)	141 (76%)	
**Laterality of ureteral reimplantation, n (%)** Left Right Bilateral	6 (13.6) 8 (18.2) 30 (68.2)	31 (22) 29 (21) 81 (57)	0.43
**ASA, n (%)** 1 2 3	3 (7) 32 (73) 9 (20)	14 (10) 117 (83) 10 (7)	0.05
BMI (kg/m^2^)	16.3 (15.3, 17.8)	16.5 (15.4, 19.1)	0.45
Operative time (minutes)	222 (186, 251)	193 (169, 229)	0.01*
EBL (ml)	10 (10, 10)	10 (10, 10)	0.01*
Postoperative narcotic use (mg/kg/day)	0.01 (0, 0.13)	0.01 (0, 0.05)	0.15
LOS (days)	2 (1, 3)	1 (1, 2)	< 0.001*
1–30 day postoperative complication, n (%)	8 (18)	14 (10)	0.18
**Clavien-Dindo grade, n (%)** 1 2 3a 3b	5 (11.4) 2 (4.5) 1 (2.1) 0 (0)	5 (3.5) 6 (4.3) 1 (0.8) 2^¥^ (1.4)	0.37
Postoperative febrile UTI, n (%)	7 (16)	29 (21)	0.66
Postoperative VCUG ^β^, n (%)	7 (16)	17 (12)	0.61
Surgical reintervention, n (%)	1 (2.3)	3 (2.2)	1
**Type of surgical reintervention, n (%)** - Subureteral injection - Other	1 0	2 1^€^	
Postoperative RBUS^£^, n Hydronephrosis status, n Stable/improved	42 42	136 136	
Follow up (months)	21 (8, 37)	16 (8, 38)	0.68

*p < 0.05

Estimates were reported as median (interquartile 1, interquartile 3) or frequency (percentage)ASA = American society of anesthesiologists; EBL = estimated blood loss; LOS = length of hospital stay; VCUG = voiding cystourethrogram; RBUS = renal bladder ultrasound

^¥^ Two required stent placement due to obstruction

^β^ No de novo or worsened reflux noted on postoperative VCUG

^€^ RALUR patient had ipsilateral obstruction requiring dismembered ureteral reimplant

^£^Two OUR and five RALUR patients did not have postoperative RBUS data available

Median follow-up duration was similar between groups (21 months [IQR 8, 37] in the OUR vs. 16 months [8, 38] in the RALUR group, *p* = 0.68). During follow-up, 7 (16%) patients in the OUR and 29 (21%) patients in the RALUR group experienced a postoperative febrile UTI (post-fUTI). The median number of post-fUTI episodes was similar between groups (OUR: 3 [IQR 2, 3] vs. RALUR: 2 [IQR 1, 2], *p* = 0.03). A total of 13 post-fUTI VCUGs were performed—3 in the OUR and 10 in the RALUR group—showing complete resolution of VUR in 10 and downgrade of reflux in 3. At the time of post-fUTI, all patients in both groups were experiencing bowel and bladder dysfunction symptoms. In terms of management post-fUTI, six patients in the OUR group were managed conservatively and one underwent a subureteral injection with Deflux^®^. In the RALUR group, 25 were managed conservatively, two required subureteral injection, one required percutaneous tibial nerve stimulation (PTNS) to help with the voiding dysfunction, and one required another surgical intervention (Table 3). The latter patient was experiencing postoperative recurrent UTI, the postop VCUG confirmed reflux resolution but a magnetic resonance urography (MRU) showed delayed kidney drainage confirming obstruction s/p dismembered reimplant. All 3 patients who underwent subureteral injection had recurrent postoperative fUTIs and persistent VUR on their postoperative VCUG. Resolution of UTI was achieved in all patients following intervention.

**Table 3:** Postoperative febrile UTI details and outcomes between open ureteral reimplant (OUR) and robot-assisted ureteral reimplant (RALUR)

	OUR	RALUR
*N* = 36^¥^	7	29
Total no. of postoperative febrile UTI	3 (2, 3)	2 (1, 2)
VCUG done post-febrile UTI, n **Results:** Resolution of VUR Downgraded to a lower VUR grade	3 2 1	10 8 2
Bowel and bladder dysfunction^Ω^, n	7	29
**Outcome after febrile UTI, n** Surveillance Subureteral injection PTNS Other	6 1 - -	25 2 1 1^β^

Estimates were reported as median (interquartile 1, interquartile 3) or frequency (percentage)

PTNS = percutaneous tibial nerve stimulation

^¥^3 patients in the OUR and 21 patients in the RALUR group had preoperative bowel and bladder dysfunction

^Ω^This entailed symptoms of urinary frequency, urgency, bladder holding habits and/or constipation reported by the parents at the time of postoperative febrile UTI

^β^Patient experienced recurrent postoperative UTI, VCUG confirmed reflux resolution and MRU confirmed delayed drainage on the left kidney s/p left dismembered reimplant due to obstruction

On multivariable regression model, there was no statistically significant difference between surgical approach and postoperative febrile UTI, while adjusting for preoperative VUR grade and age at surgery (OR = 1.67, 95% CI:0.63–4.45, *p* = 0.30) (Table 4).

**Table 4:** Multivariable logistic regression model looking at the association of surgical approach and febrile UTI while adjusting for reflux grade and age at surgery

Variable	Odds Ratio	95% Confidence Interval	*p* Value
**Surgical approach** OUR RALUR	Reference 1.67	- 0.63–4.45	0.30
Age at surgery (years)	0.89	0.79-1	0.05
**VUR grade** Nondilating (Gr I-II) Dilating (Gr III-V)	Reference 0.30	- 0.10–0.87	0.03

## Discussion

This study aimed to present a single institution’s experience and compare outcomes between RALUR and OUR. In a modern cohort of patients undergoing surgery for primary VUR, although we found differences in age at surgery and grade of reflux, there were no differences in the rate of febrile UTIs, post-procedure complications or need for re-intervention during follow-up. Controlling for those patient characteristics, additionally found no association between surgical approach and an increased risk of post-procedure febrile UTI.

The comparison between open and minimally invasive anti-reflux surgery has been a topic of interest for many years. Smith et al. conducted a retrospective age-matched comparative study between OUR and RALUR, reporting an overall success rate of 97% for RALUR and 100% for OUR [7]. Sforza et al. published a multi-institutional study comparing open versus robot-assisted ureteral reimplantation in children with high grades of reflux (IV-V), reporting success rates of 94% and 98.5% in the open and RALUR groups, respectively, with comparable long-term clinical success [15]. Marchini et al. reported a case-matched comparative study between OUR and RALUR, concluding that there was no difference between the two approaches for clinical or radiological success [10].

The primary treatment goal is to reduce postoperative febrile UTIs. Our study revealed that 20.8% of patients in the RALUR and 17.8% in the OUR groups experienced postoperative febrile UTIs. Although higher than historical reports, in the post-RIVUR trial [16], similar rates have been reported in the literature, with more recent studies indicating febrile UTI incidence after anti-refluxing surgery ranging from 8% to 24% [17–21] Nelson et al. reported an overall incidence of 21.8% postoperative febrile UTI in their retrospective study of open ureteral reimplantation [22]. Seungsoo et al. documented that 31.3% of their cohort experienced postoperative febrile UTI after OUR [23]. Additionally, Brandstrom et al. conducted a randomized controlled trial involving 203 children across 23 centers, reporting a 23% postoperative febrile UTI rate with endoscopic therapy alone [24]. However, a multi-institutional study comparing outcomes of Laparoscopic Ureteral Reimplant (LUR) versus RALUR-EV across 5 centers reported a 0% postoperative febrile UTI rate after RALUR-EV [25]. This heterogeneity in outcomes is not explained by technical factors alone. Many factors such as duration of follow-up, appropriately determining if a fUTI occurred, pre- and post-operative emphasis on urotherapy (i.e. hydration, bladder emptying, preventing constipation) likely all account for these reported differences.

In addition to post-procedure febrile UTIs, the rate of complications between these two approaches has generated much discussion. Previously published large, database studies, have shown increased rates of complications in patients undergoing RALUR compared to OUR [11, 26]. Those experiences likely reflected early experiences in pediatric robotic surgery as our recent cohort showed no major difference in the incidence or grade of Clavien-Dindo [14] complication experienced within the first 30 days post-procedure. Like many novel surgical procedures, as safety has improved so has efficiency and outcomes.

In cohort of patients, Sahadev et al. reported that RALUR offers several benefits over the open approach, including improved field of vision via intraperitoneal magnification, enhanced cosmesis for the patient, lower incidence of bladder spasm or hematuria and faster recovery due to an extravesical approach [9].

In 2004, Peters was the first to report RALUR in the pediatric population, with reflux correction occurring in 89% of the cohort [4]. Later in 2005, Peters and Woo presented a case series of intravesical RALUR, showing resolution of reflux in 83% of patients, with one experiencing a urine leak as a postoperative complication [27]. In 2008, Casale et al. reported outcomes in 41 patients undergoing RALUR, with an overall surgical success rate of 97.6% [12].

Mittal et al. published a retrospective study on RALUR via an extravesical approach (RALUR-EV), reporting a 23% febrile UTI incidence at any point in extended follow-up in their cohort [20]. Akhavan et al. retrospectively reviewed patient outcomes after RALUR, reporting a 10% postoperative febrile UTI rate [21].

The observed differences in age at surgery between patients with low and high grade reflux reflects the natural history of VUR. Patients with lower-grade reflux were more likely to be managed nonoperatively earlier in life and underwent surgical intervention only after development of febrile UTIs with persistent reflux, in most cases supported by evidence of renal scarring on imaging.

This study has several limitations. First, its retrospective, single-institution design may introduce selection bias and limit the generalizability of our findings to other institutions with varying surgical techniques and patient populations. Second, although we aimed to compare long-term outcomes between RALUR and OUR, our median follow-up period was limited to approximately 17–21 months, which may not fully capture late complications. Furthermore, postoperative voiding cystourethrogram (VCUG) was not routinely performed in all patients and was instead used selectively in cases of recurrent postoperative febrile UTIs or ongoing concern for persistent reflux, potentially leading to an underestimation of radiographic recurrence rates. Instead, clinical success was primarily assessed through postoperative febrile UTI rates, which, while meaningful, may not provide a complete picture of reflux resolution. Another limitation is the lack of standardized criteria for assessing voiding dysfunction, which was based on parental observations rather than objective studies. This could introduce variability in identifying voiding dysfunction as a predisposing factor for persistent or recurrent UTIs.

## Conclusion

In conclusion, our study demonstrates comparable outcomes between robot-assisted laparoscopic ureteral reimplantation (RALUR) and open ureteral reimplantation (OUR) in pediatric patients with primary vesicoureteral reflux. Despite differences in patient demographics and perioperative characteristics, both surgical approaches yielded similar rates of postoperative febrile urinary tract infection (UTI) and overall complication rates. These findings support the feasibility and effectiveness of RALUR as a minimally invasive alternative to traditional open surgery for managing vesicoureteral reflux in children.

## Data Availability

The data that support the findings of this study are available from the corresponding author upon reasonable request and following the establishment of a data-sharing agreement.
